# Miniaturized and Actively Tunable Triple-Band Terahertz Metamaterial Absorber Using an Analogy I-Typed Resonator

**DOI:** 10.1186/s11671-022-03677-5

**Published:** 2022-03-15

**Authors:** Ben-Xin Wang, Chongyang Xu, Guiyuan Duan, Jieying Jiang, Wei Xu, Zhuchuang Yang, Yangkuan Wu

**Affiliations:** School of Science, Jiangnan University, Wuxi, 214122 Jiangsu China

**Keywords:** Terahertz metamaterial, Perfect absorber, Triple-band absorption, Analogy I-typed resonator

## Abstract

Triple-band terahertz metamaterial absorber with design of miniaturization and compactness is presented in this work. The unit cell of the terahertz absorber is formed by an analogy I-typed resonator (a rectangular patch with two small notches) deposited on top of dielectric sheet and metallic mirror. The miniaturized structure design exhibits three discrete frequency points with near-perfect absorption at terahertz regime. The three absorption peaks could be ascribed to localized resonances of analogy I-typed resonator, while the response positions of these absorption peaks at the analogy I-typed resonator are different by analyzing the near-field patterns of these resonance peaks. Changes in structure parameters of the analogy I-typed resonator are also investigated. Simulation results revealed that the notch sizes of the rectangular patch are the key factor to form the triple-band near-perfect absorption. Further structure optimization is given to demonstrate triple-band polarization insensitive performance. Moreover, actively tunable absorption properties are realized by inserting or introducing vanadium dioxide with adjustable conductivity into the metamaterial structure. It is revealed that the insulator–metal phase transition of vanadium dioxide is the main reason for the modulation of absorption performance. Compared with previous multiple-band absorbers, the device given here has excellent features of high degrees of simplification, miniaturization, and active modulation, these are important in practical applications.

## Introduction

Metamaterials are engineered (or artificial) materials formed by sub-wavelength scales of ordered or disordered building blocks, which could be utilized to achieve novel and extraordinary electromagnetic phenomena that natural materials cannot possess [1–3]. Over the past decade, research on metamaterials has shifted from original theoretical analyses to practical application areas of functional devices [4–13], including sensors, filters, modulators, absorbers, etc. In these devices, metamaterial-based light absorbers (MBLAs) using structures of metal-dielectric-metal have attracted rapidly growing research attention because they have the ability to achieve near 100% (or 100%) absorption with an ultra-thin thickness of middle dielectric sheet. These two outstanding features (large absorption strength and ultra-thin dielectric sheet thickness) are of considerable importance in practical applications. This is why we have witnessed the rapid development of MBLAs [14–18].

However, these MBLAs with single-band absorption characteristics have great limitations in many applications. The designs of multiple-band and broadband MBLAs are quite urgent. Normally, the increases in the absorption peaks (or the broadening of resonance bandwidths) of the MBLAs frequently utilize the design methods of multiple-layer stacked structure and coplanar super-unit structure [18–33]. Although the two methods can enable multiple-band or broadband absorption, results obtained in these references possess common characteristic that demands several elements or building blocks with different sizes. For example, three, four, eight, and even nine components were reported in Refs. [24–33] to achieve triple-band MBLAs. Obviously, these designs undoubtedly possess large lattice dimensions, time-consuming and laborious manufacturing steps.

Very recently, some structure designs by reducing the number of the resonators have been suggested to obtain the triple-band absorption. For example, unit cells of Refs. [34, 35] consisted of two resonators were designed to realize triple-band MBLAs. Further structure optimization of triple-band absorption device was reported using a snowflake-shaped resonator with strip spiral line load [36]. These approaches, while can reduce the number of resonators, introduce new issues. Firstly and most importantly, these designs are quite complex in themselves because they possess considerable number of geometric parameters, which will inevitably increase the optimization time. Secondly, these triple-band MBLAs can only operate in the microwave frequency range, which are difficult to play a practical role in other frequency bands. Taking into account the above two factors, therefore, using simple and less number of structure designs to achieve triple-band MBLAs operating in other promising spectral regions (such as terahertz frequency band) are still challenging.

In this work, a single-sized resonator with simple structure design is suggested to achieve triple-band absorption at terahertz frequency. The device composes of an analogy I-typed resonator (actually is a rectangular patch with two small notches) deposited on top of a dielectric sheet, which is backed by a metallic board. Three near-perfect terahertz discrete absorption points are realized in this simple single-sized resonator, and the basic principle of them is caused by three different localized resonances, while the near-field response positions of them are different. It is further proved that the structure sizes of the two small notches are important in determining the number of the absorption points. Additionally, triple-band polarization insensitive terahertz MBLA is given by reasonable optimization of a square patch with four small notches. In sharp contrast to previous demonstrations, the devices presented here provide the ability to simultaneously obtain triple-band terahertz absorption and high degree of simplification, and therefore the designed platforms have great application potentials in terahertz technology related areas. The comparison with the previous triple-band metamaterial absorbers is shown in Table 1, which can intuitively show the advantages of this manuscript in terms of construction method, design strategy, resonance performance, etc.

**Table 1:** Comparison between the designed triple-band absorber and previously reported triple-band metamaterial absorbers

References	Number of absorption peaks	Number of sub-resonators	Polarization tolerance	Active modulation	Working region	Main materials	Fabrication steps
[37]	3	3	No	Yes	Mid-infrared	Graphene	Rather Difficult
[38]	3	2	Yes	No	Microwave	Copper	Easy
[39]	3	3	Yes	No	Microwave	Copper	Easy
[40]	3	5	Yes	No	Microwave	Copper	Easy
[41]	3	4	No	Yes	Terahertz	Graphene	Rather Difficult
[42]	3	6	Yes	No	Terahertz	Gold	Easy
[43]	3	3	Yes	No	Microwave	Copper	Easy
[44]	3	3	Yes	No	Microwave	Copper	Easy
[45]	3	6	Yes	No	Microwave	Copper	Easy
[46]	3	5	Yes	No	Near-infrared	Gold	Difficult
[47]	3	3	Yes	No	Terahertz	Aluminum	Easy
[48]	3	3	Yes	No	Mid-infrared	Gold	Difficult
[49]	3	5	Yes	Yes	Terahertz	Gold	Easy
[50]	3	5	No	Yes	Terahertz	VO_2_	Easy
[51]	3	3	Yes	No	Terahertz	Aluminum	Easy
[52]	3	3	No	No	Terahertz	Gold	Easy
This work	3	1	Yes	Yes	Terahertz	Gold	Easy

## Structure and Design

**Fig. 1:**
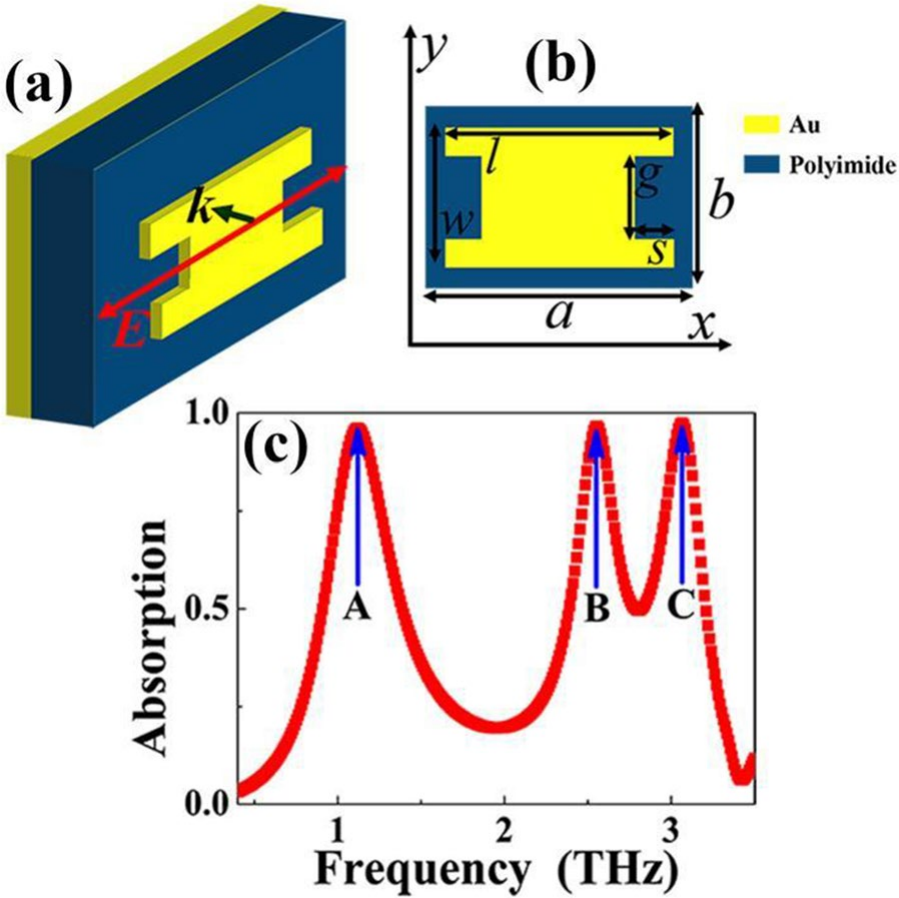
**a** Three-dimension structure sketch of the triple-band MBLA, of which the red line with arrow indicates the polarization direction of incident electromagnetic wave, the black line with arrow gives the propagation direction of incident electromagnetic wave; **b** Front view of the triple-band MBLA; **c** Simulated absorption spectra of the designed device as a function of frequency

The three-dimension sketch of the presented MBLA is illustrated in Fig. 1a. It is formed by an Au resonator deposited on top of a dielectric sheet with thickness of 14 μm while bottom layer is backed by a continuous Au board. The choice of the dielectric sheet is polyimide [53, 54], here its refractive index is set to 1.73 + *i*0.1, the two Au layers have conductivity of 4.09 × 10^7^ S/m. Figure 1b exhibits the front view of the MBLA. As given, the Au pattern can be considered as an analogy I-typed resonator, which is actually a rectangular patch with two small notches, where the patch has the length (*l*) and width (*w*) of 70 μm and 55 μm, respectively, the small notch, respectively, has the length (*s*) and width (*g*) of 12 μm and 40 μm. Please note that the ratio of the middle line-width (*l − *2*s*) and patch length (*l*) is about 65.71%, which is much greater than the conventional I-typed resonator [55–57], thus we call it the analogy I-typed resonator. The basic unit cell has the dimensions of *a* = 88 μm and *b* = 68 μm. A numerical method based on finite difference time domain algorithm (using the software of FDTD Solutions, version 8.6) is utilized for analyzing and studying the designed MBPLA. We choose a plane wave polarized along the *x*-axis to irradiate the unit cell. Because the unit cell is infinite extension in both directions of *x* and *y*, periodic boundary conditions are applied along the two axes. Additionally, scattering should be avoided in light propagation direction, and therefore perfectly matched layers are assigned in the *z*-axis.

The possible experimental fabrication steps are as follows: Firstly, a layer of continuous gold film can be evaporated onto a substrate (such as silicon or quartz crystal) as a reflective layer. Because the thickness of continuous gold film is greater than the skin depth of light beam, the choice of substrate materials usually does not affect the absorption property of metamaterial absorber. Secondly, a polyimide layer could be spin-cast as the dielectric sheet for the metamaterial absorber, and its thickness could be modified by controlling the spin speed or duration. Electron beam lithography could be used as a suitable method for defining the metallic pattern layer. Finally, a layer of gold film could be evaporated again, and the pattern transfer could be completed by metal lift-off [58, 59].

## Results and Discussion

**Fig. 2:**
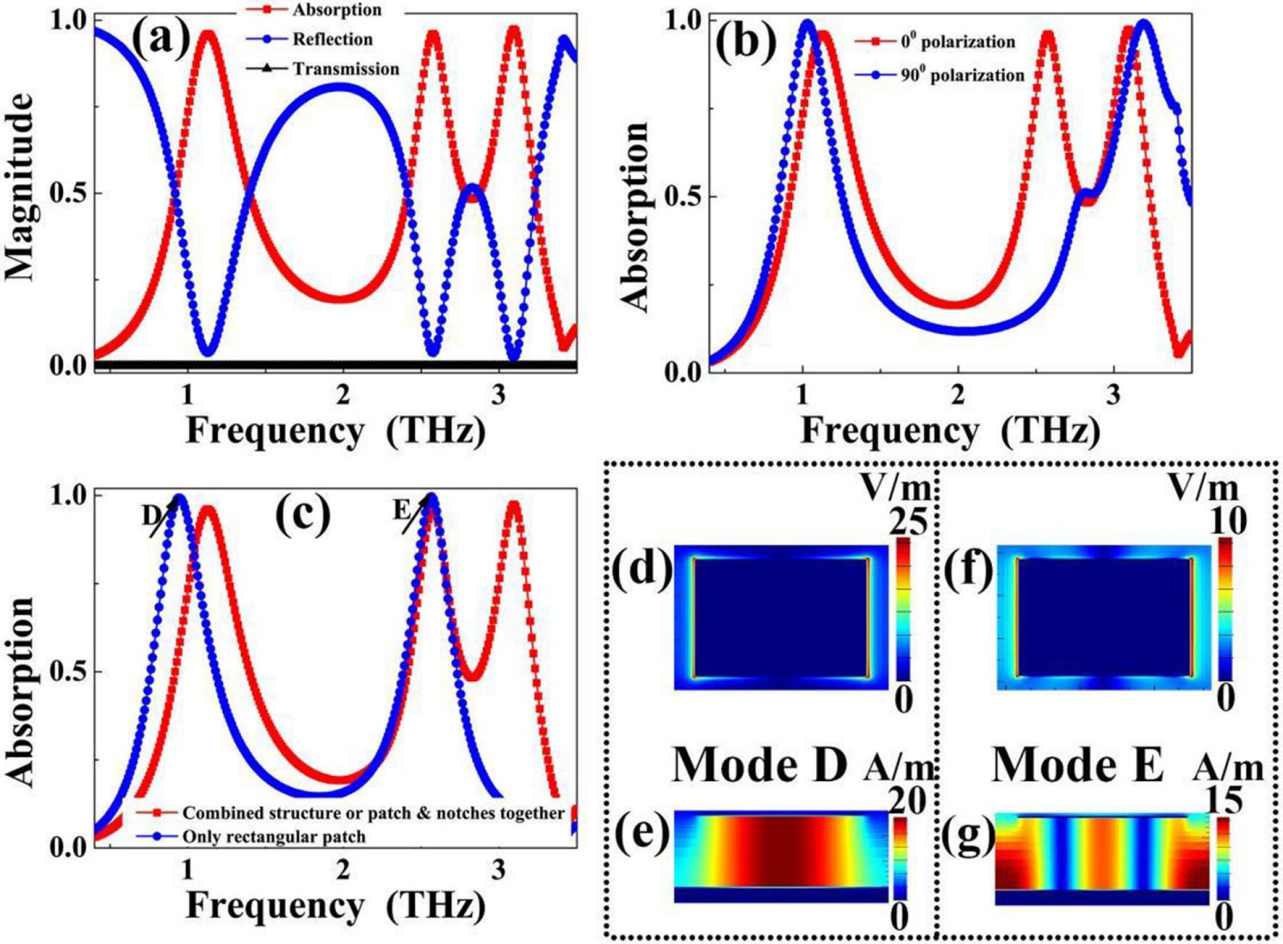
**a** Absorption (red line), reflection (blue line) and transmission (black line) of the designed triple-band MBLA; **b** simulated absorption spectra of designed device as a function of frequency under two orthogonal polarizations (0° means that the polarization is along the *x* axis, while 90° means that the polarization is along the *y* axis); **c** comparison of absorption properties of designed device (i.e., the combined structure or patch & notches together or analogy I-typed resonator) and the rectangular patch absorber; **d** electric field |*E*| distribution of mode D for rectangular patch absorber with the blue line at **c**; **e** magnetic field |*Hy*| distribution of mode D for rectangular patch absorber with the blue line at **c**; **f** electric field |*E*| distribution of mode E for rectangular patch absorber with the blue line at **c**; **g** magnetic field |*Hy*| distribution of mode E for rectangular patch absorber with the blue line at **c**

The simulated absorption spectra of the suggested resonant device (analogy I-typed resonator) as a function of frequency is described in Fig. 1c. The absorption (*A*), transmission (*T*) and reflection (*R*) of the designed structure under same frequency ranges are also presented, as shown in Fig. 2a, to facilitate the comparison of optical results. The absorption *A* is given by *A* = 1 − *T* − *R*. Near-perfect absorption (or *A* ≈ 100%) could be achieved by completely suppressing the *T* and *R*. From the results in Figs. 1c and 2a, we can see that that the analogy I-typed resonator exhibits three near 100% absorption resonance points centered at *A* = 1.12 THz, *B* = 2.58 THz, and *C* = 3.09 THz. The FWHM (full width at half maximum) for frequency point A is about 0.49 THz, and its corresponding *Q* (quality factor) is about 2.29. The *Q* is defined based on formula [60]:

1 $$Q = f/{\text{FWHM}}$$

of which the *f* is the resonance frequency of absorption peak. The frequency point B has the FWHM of 0.33 THz and *Q* of 7.76. The FWHM and *Q* of the frequency point C are, respectively, 0.40 THz and 7.68. The three near-perfect frequency points are all originated from the localized responses of the analogy I-typed resonator, while their near-field aggregation regions or positions on the analogy I-typed resonator are different. The detailed analyses are discussed in the following paragraphs. Compared with previous triple-band MBLAs, see Table 1, the device given here has the features of high degrees of simplification, miniaturization, active modulation and so on. This is what is really required in practical applications. However, the obtained absorption performance shows an obvious dependence on polarization angles due to anti-symmetric surface structure (analogy I-typed resonator) of the triple-band metamaterial absorber, as shown in Fig. 2b. Actually, slight modification of the surface structure (forming a high degree of symmetry) can make its performance insensitive to change of polarization angles, see below Fig. 5.

In order to intuitively understand and preliminarily explore the formation mechanism of triple-band absorption, we give the comparison of the absorption features of designed device having the surface structure of combined pattern (or patch & notches together or analogy I-typed resonator) and the rectangular patch. As shown by the blue line of Fig. 2c, the rectangular patch has two absorption peaks, of which the first one is marked as mode D and the second one is marked as mode E. The resonance frequencies of modes D and E are 0.93 THz, and 2.57 THz, respectively. More importantly, we found that the mode E basically coincides with the second absorption peak (mode B) of the combined pattern (analogy I-typed resonator). From this point, it can be roughly inferred that the formation mechanisms of the two absorption peaks (mode E of rectangular patch and mode B of analogy I-typed resonator) seems to be consistent. In fact, the latter electromagnetic field distributions and analyses can confirm this inference.

It can be further confirmed that the formation mechanisms of mode D of rectangular patch and mode A of the analogy I-typed resonator is consistent by analyzing their electromagnetic field distributions. The inconsistency between their resonance frequencies is mainly reflected in the difference of effective length of surface patterned structure of the designed metamaterial absorber. When the designed metamaterial absorber, especially its surface structure, interacts with the incident electromagnetic wave, the (effective) length of rectangular patch with two notches (or analogy I-typed resonator) should be less than that of the rectangular patch without notches. According to the *LC* circuit model [61–63], the longer the (effective) length is, the smaller the resonance frequency is. Therefore, the resonance frequency of mode A of the analogy I-typed resonator should be greater than that of absorption peak D of rectangular patch, which is consistent with the theoretical calculation, see Fig. 2c.

Therefore, to explore formation mechanism of the triple-band absorption, it is necessary to clarify the physical origin of absorption peaks D and E of the rectangular patch because the generation of absorption peaks A and B of triple-band metamaterial absorber is very closely related to them. Actually, the absorption peaks D and E of the rectangular patch absorber are mainly derived from the first-order and third-order localized resonance responses of the rectangular patch. This judgment is mainly based on the following two main reasons:

1. Firstly, the electric field distributions of modes D and E are mainly focused on the edges of rectangular patch, and their magnetic fields are chiefly distributed at the dielectric sheet of the metamaterial absorber, as shown in Fig. 2d–g. These near-field distribution features indicate that the absorption peaks D and E are both attributed to localized resonance responses of rectangular patch. Moreover, for magnetic field distribution in Fig. 2e, there is an obvious aggregation region (or node) for the absorption peak D, while three obvious aggregation regions (or nodes) are observed for absorption peak E in Fig. 2g. The magnetic field distributions of Fig. 2e, g revealed that the modes D and E should be derived from the first-order and third-order localized resonance responses of the rectangular patch, respectively. This is because the accumulation regions and features of their near-field distributions, especially the magnetic field figures, completely accord with the near-field aggregation properties of the corresponding orders [64–69].

2. Secondly, based on *LC* circuit model and theory of classical patch antenna [61–63], the resonance frequency (*f*_*i*_) of the metamaterial absorber can be generally expressed as:

2 $$f_{i} \approx (2i - 1)c/2nl$$

in which *c* is light speed, *n* is refractive index of dielectric sheet, *l* is (effective) length of the surface structure of designed metamaterial absoreber, *i* is an integer (where *i* = 1, 2, 3…). For Eq. (2), the resonance frequency of metamaterial absorber decreases gradually with the increase of (effective) length of its surface structure. More importantly, the resonance frequency of third-order (*i* = 2) response of the surface structure of the metamaterial absorber should be three times that of the firs-order (*i* = 1) response. According to the absorption curve of the rectangular patch in blue line of Fig. 2c, the frequency of mode *E* (2.57 THz) is 2.76 times that of mode *D* (0.93 THz), approximately three times. Therefore, the modes D and E should be ascribed to the third-order and first-order resonance responses of the rectangular patch, respectively. The reason why the ratio of the two frequencies is slightly less than three times may be due to the interaction between the units. It is found that there are some magnetic field distributions at the edges of the dielectric sheet and some small proportion of electric fields are observed at the boundaries of the unit, see Fig. 2f, g.

**Fig. 3:**
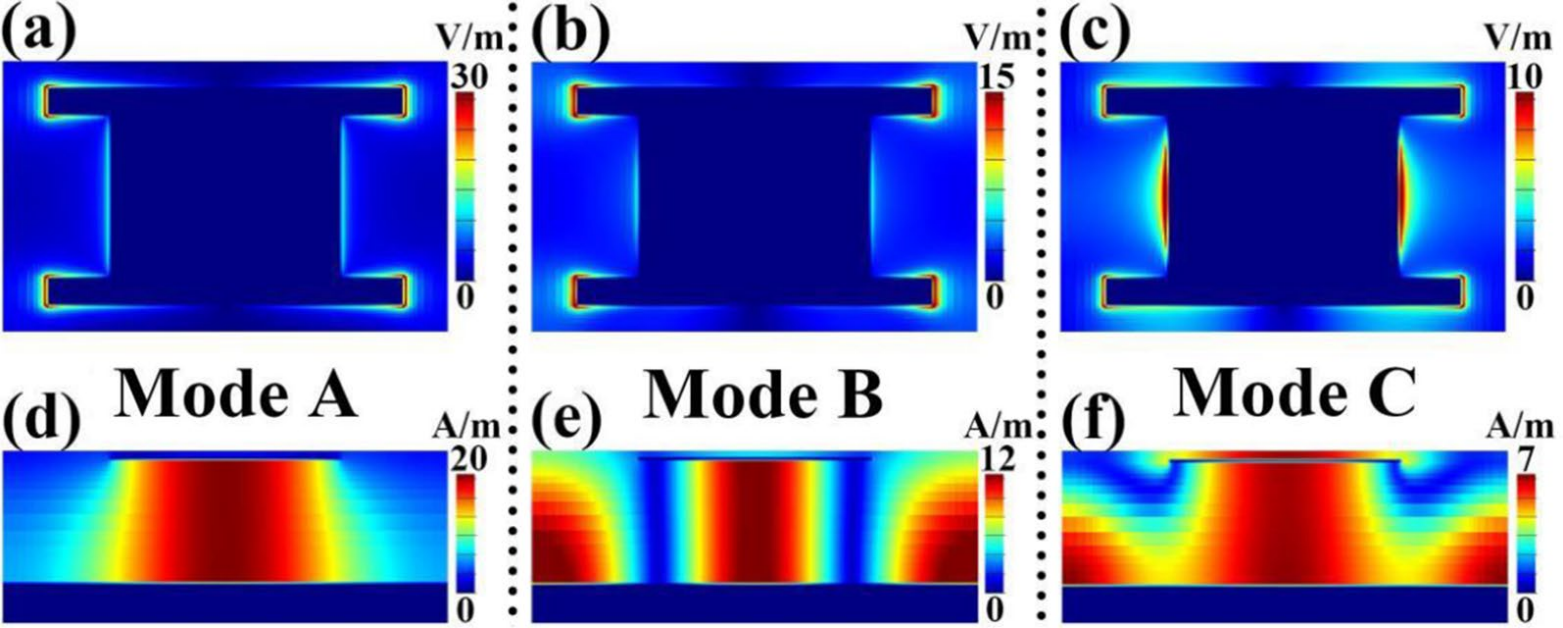
**a**–**c** provide the |*E*| field distributions of frequency points A, B, and C of the analogy I-typed resonator, respectively; **d**, **e**, and **f** provide the |*Hy*| field distributions of frequency points A, B, and C of the analogy I-typed resonator, respectively

Since the formation mechanisms of absorption peaks D and E of the rectangular patch have been confirmed, the physical pictures of absorption peaks A and B of the analogy I-typed resonator can be easily known. To better understand and clarify the cause of the triple-band absorption, the near-field distributions of the three frequency points (A, B, and C) are presented in Fig. 3, of which (a), (b), and (c) give the electric (|*E*|) field distributions of the modes A, B, and C in the plane that intersects the middle height of the analogy I-typed resonator, respectively, and the magnetic (|*H*y|) field distributions of the modes A, B, and C (in the plane that is parallel to the light propagation direction) are provided in (d), (e), and (f), respectively. As observed in Fig. 3, the |*E*| field distributions of the three frequency points are mostly distributed over the edges of the analogy I-typed resonator, meanwhile, the dielectric sheet of the triple-band MBLA provides the space for the |*H*y| field aggregations of the three absorption modes, indicating the absorption peaks A, B, C are all attributed to the localized responses of suggested metamaterial absorber. However, the near-field aggregation and enhancement regions (or positions) of the three absorption peaks on the analogy I-typed resonator and dielectric sheet are different.

For frequency point A, its electric (|*E*|) field is symmetrically focused on both sides (especially the corners) of the analogy I-typed resonator, see Fig. 3a, which means that the same but opposite charges will be generated at the edges of analogy I-typed resonator when the incident electromagnetic wave interacts with the resonator. The existence of the same but opposite charges will excite the dipole resonance mode on the analogy I-typed resonator [64–69]. At the same time, the bottom continuous metallic mirror of the designed metamaterial absorber will induce a current having the opposite direction to the surface structure (analogy I-typed resonator) [64–69]. The existence of opposite currents between the top and bottom metallic layers will induce magnetic resonance, thus an obvious magnetic field aggregation effect can be obtained in the dielectric sheet of metamaterial absorber [64–69]. As observed in Fig. 3b, the magnetic (|*H*y|) field distribution of the absorption peak A is indeed strongly gathered in the dielectric sheet, which verifies the correctness of the theoretical analysis. We further found that the near-field (electric and magnetic) distributions of the absorption peak A are very similar to that of the absorption peak D in Fig. 2d, e. In particular, its magnetic (|*H*y|) field with single strong aggregation area (or node) in the dielectric sheet is observed. All the near-field distribution characteristics of absorption peak A in Fig. 3a, d illustrate that this absorption mode should be caused by the first-order resonance response of analogy I-typed resonator [64–69].

For absorption peak B, it is observed that its electric (|*E*|) field in `. 3(b) can be intensely centered on both sides (especially the corners) of the analogy I-typed resonator, which is very similar to that of the frequency point A in Fig. 3a. But the magnetic (|*H*y|) field distributions of the two frequency points are different, which are mainly reflected in the number of field aggregation and enhancement regions (or nodes). Only single strong aggregation region (node) is observed in Fig. 3d, while three strong aggregation regions (or nodes) can be clearly found in Fig. 3e, which shows that frequency point B stems from the third-order resonance response of the analogy I-typed resonator of triple-band metamaterial absorber [64–69].

For frequency point C, neither the electric (|*E*|) field in Fig. 3c nor the magnetic (|*H*y|) field in Fig. 3f is different from the cases of the first two frequency points A and B. More concretely, the electric (|*E*|) field of frequency point C in Fig. 3c is mainly concentrated not only on the four corners of the analogy I-typed resonator, but also both edges of middle part of the analogy I-typed resonator. Particularly, the percentage of the electric field distribution in both edges of the middle part of the resonator is greater than that of the four corners of the analogy I-typed resonator. Furthermore, its magnetic (|*H*y|) field in Fig. 3f is filled with the whole dielectric sheet, which is different from the cases of the first two modes A and B that certain number of the field aggregation regions (or nodes) can be obtained in the dielectric sheet, see Fig. 3d, e. The near-field distributions of the frequency point C indicate that this mode should be caused by the hybridization effect of localized resonance responses in the corners and middle part of the analogy I-typed resonator. On the basis of the discussion above, the combining effect of three localized resonances having different near-field aggregation and enhancement positions results in the triple-band near-perfect absorption. Compared with previous strategies to achieve triple-band absorption, see Table 1, the method presented here has only single resonator with rather simple structure design and excellent resonance performance, paving the way for the design of multiple-band (especially triple-band) integrated terahertz functional absorption devices.

**Fig. 4:**
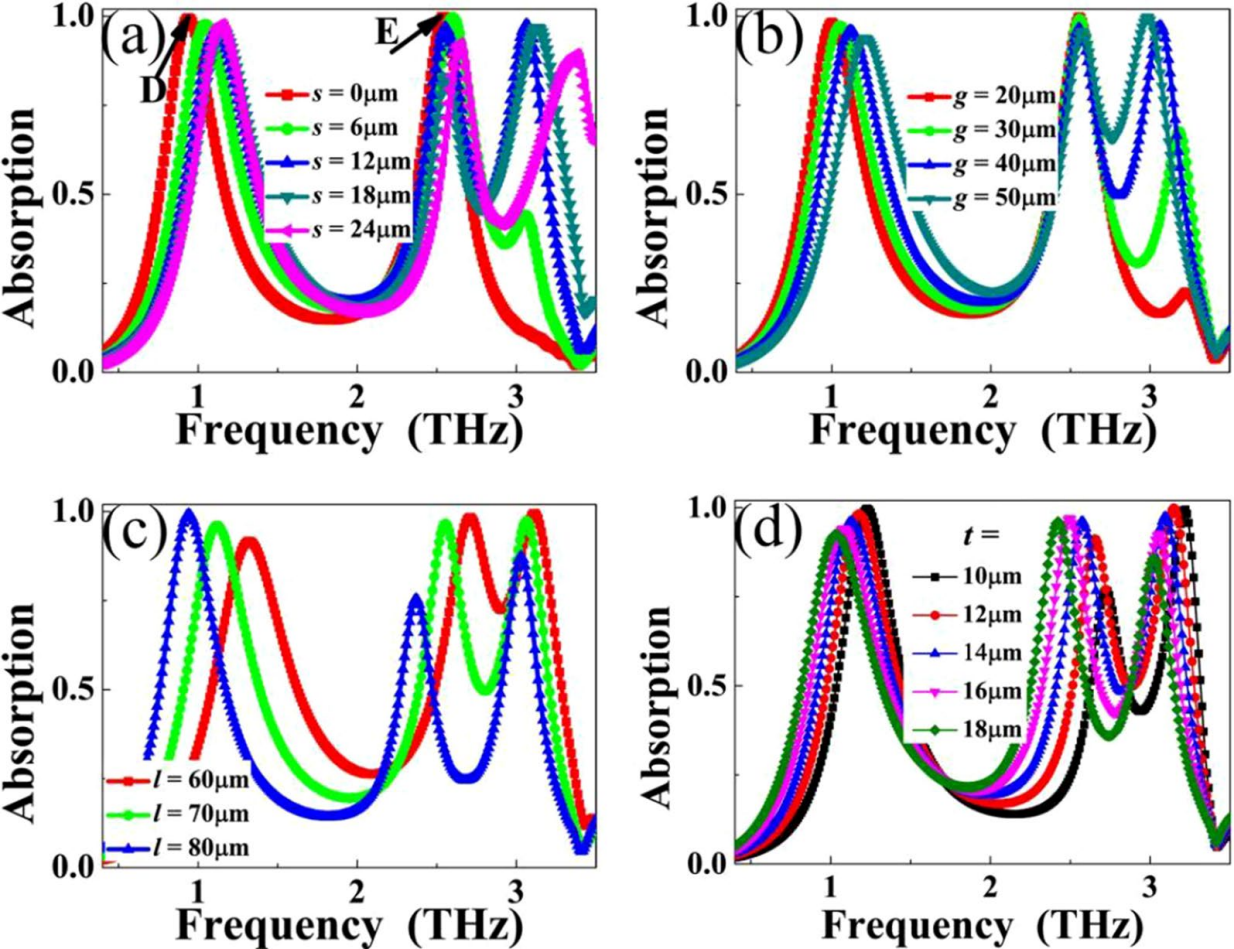
Dependence of absorption spectra as a function of frequency on the parameter changes of **a** notch length *s*; **b** notch width *g*; **c** entire resonator length *l*; **d** dielectric sheet thickness *t*

In the analysis of the resonance mechanisms of the triple-band metamaterial absorber, the electric (|*E*|) field patterns of the three frequency points are all highly gathered in both edges of the analogy I-typed resonator, indicating the length (*l*) change of metallic resonator should intensely affect their resonance frequencies. As observed in Fig. 4c, the three frequency points are indeed depended on the resonator length *l*. According to Eq. (2), we all know that the metamaterial absorber frequency is inversely proportional to resonator length. Results of Fig. 4c clearly present such resonance feature that the frequencies of the three points gradually decrease with the *l* increase. Furthermore, it is observed that the size changes of the two small notches in the rectangular patch also affect the performance of the absorption device, in particular of the third frequency point. As shown in Fig. 4b, the frequency point C is nearly suppressed for the notch width *g* = 20 μm. The frequency point C can be completely suppressed when the analogy I-typed resonator is changed to rectangular patch (i.e., notch length *s* = 0), see Fig. 4a. In other words, the rectangular patch can enable dual-band absorption centered at *D* = 0.93 THz and *E* = 2.57 THz. Results shown here prove that the notch sizes in the rectangular patch are the key factor to realize the triple-band absorption. Additionally, we further found that the thickness changes of the dielectric sheet also have important impact on the absorption properties, see Fig. 4d. This is mainly because the variation of the dielectric sheet thickness could change the (effective) refraction index and impedance of whole triple-band metamaterial absorber, therefore the resonance frequencies and absorption rates of the designed metamaterial absorber can be modulated, respectively.

Here we would like to give some brief explanations for why the frequency blue shifts with the increases of *s* and *g,* and reduce (or even suppress) the absorption intensity of peak C with the decreases of *s* and *g*. Firstly, no matter increasing *s* or *g*, it will indirectly reduce the (effective) length of the surface structure, the reduction of the (effective) length of the surface structure will inevitably increase the resonant frequency of the corresponding absorption peak, that is, the blue shift phenomenon occurs, see Fig. 4a, b. This is opposite to the change trend of absorption frequency with the increase of length *l* in Fig. 4c. Secondly, with the decreases of *s* and *g*, the designed analogy I-typed resonator gradually transits to rectangular patch, so the absorption intensity of peak C can be expected to be decreased or suppressed. In other words, the smaller sizes of two notches in rectangular patch could not provide enough electromagnetic enhancement effect to provide the possibility of achieving near-perfect absorption of peak C. There is no doubt that for extreme case (rectangular patch), that is, without two notches, the absorption peak C will be completely suppressed, as shown in blue line of Fig. 2c and the red line of Fig. 4a.

**Fig. 5:**
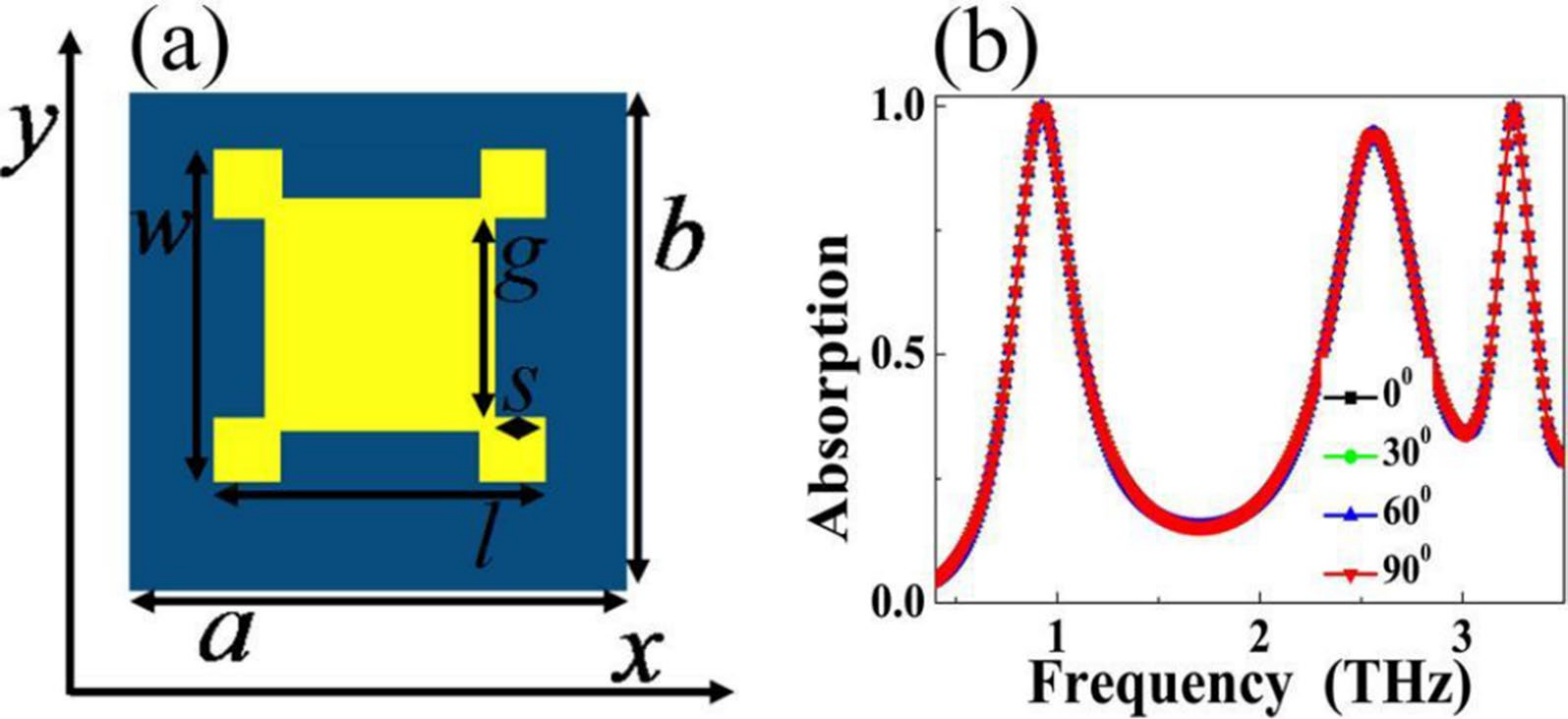
**a** Front view of the triple-band polarization insensitive terahertz MBLA; **b** Absorption spectra of the designed device as a function of frequency

The suggested triple-band MBLA has the absorption feature of polarization sensitive because of the asymmetric resonance structure, see Fig. 2b. For many applications, however, the absorption performance of polarization insensitive is required. Here we further optimize the structure design to demonstrate a triple-band polarization insensitive terahertz MBLA. Its basic cell is consisted of three layers (two Au layers separated by a dielectric sheet), which is very similar to that of the unit structure in Fig. 1a. However, the front view of the triple-band polarization insensitive MBLA is different from the case of the polarization sensitive in Fig. 1b. It is formed by a square Au patch with four same-sized notches, see Fig. 5a. The square patch has the length of *l* = *w* = 70 μm. The notch has the length of *s* = 12 μm, width of *g* = 20 μm. Repeat periods of the basic unit cell in both directions of x- and y-axis are respectively *a* = 88 μm, and *b* = 88 μm. The thickness and refractive index of the dielectric sheet, the conductivity of the two Au layers as well as the boundary conditions of the model are the same as the suggested device in Fig. 1. The absorption spectra of the structure design of Fig. 5a is depicted in Fig. 5b. As given, in different polarization angles, such as 0°, 30°, 60° and even 90°, three near 100% absorption frequency points centered at 0.93 THz, 2.56 THz, and 3.25 THz are achieved, indicating the suggested structure in Fig. 5a can enable the triple-band polarization insensitive resonance performance, which should be ascribed to the high degree of symmetry of the resonance structure.

## Active Modulation Performance

It is quite easy to achieve active modulation of absorption properties by inserting or introducing some materials with variable conductivity into the initially designed metamaterial structure. Vanadium dioxide (VO_2_), as a typical phase change material, its dielectric permittivity undergoes a reversible insulator–metal phase transition around 340 K. This transition can be achieved by changes in some forms of stimulation, such as light, heat, stress, etc. As a promising candidate for optoelectronic functional materials, the conductivity (*σ*) of VO_2_ could be varied by 4–5 orders of magnitude, and its phase transition can be completed in order of picosecond. The optical permittivity of VO_2_ at terahertz frequency region can be described by Drude model, please see Refs. [70–72] for details. Therefore, VO_2_ possesses wide application prospects in tunable or reconfigurable metamaterial resonators. To obtain the tunable absorption properties, VO_2_ is inserted or introduced into the triple-band metamaterial absorber structure to explore its active modulation features. In this section, two kinds of design strategies are considered to realize active modulation of the triple-band absorption.

**Fig. 6:**
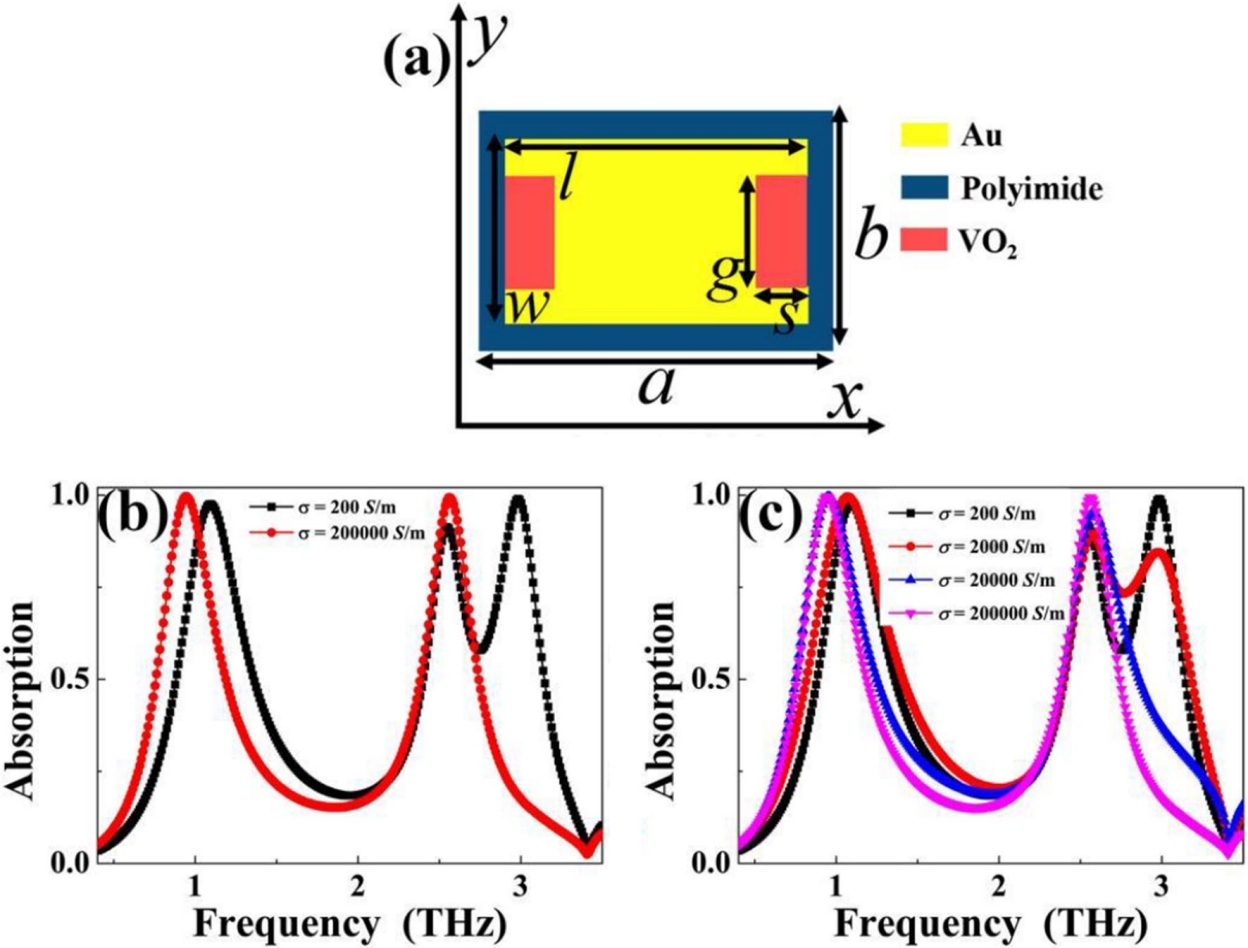
**a** Front view of tunable metamaterial absorber (case 1) by inserting VO_2_ into two notches; **b** Absorption curves of tunable terahertz metamaterial absorber (case 1) under conductivity values of *σ* = 200 S/m and *σ* = 200,000 S/m; **c** Absorption curves of tunable metamaterial absorber (case 1) under conductivity values

**Fig. 7:**
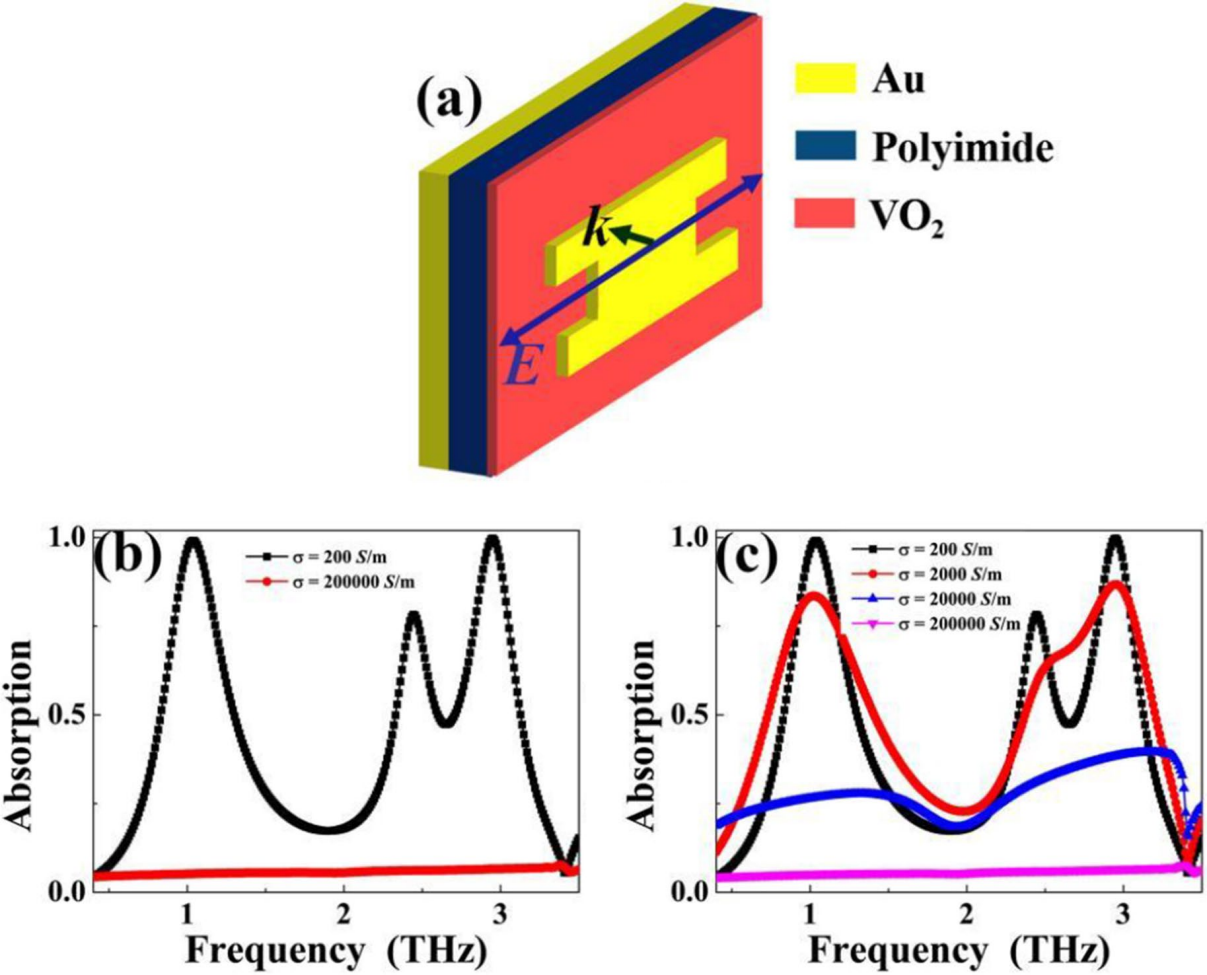
**a** Three-dimension structure sketch of tunable metamaterial absorber (case 2) by introducing VO_2_ continuous layer with the thickness of 0.4 μm under the layer of analogy I-typed resonator; **b** absorption curves of tunable terahertz metamaterial absorber (case 2) under conductivity values of *σ* = 200 S/m and *σ* = 200,000 S/m; **c** absorption curves of tunable metamaterial absorber (case 2) under conductivity values

(1) The first modulation strategy is to insert VO_2_ into two notches (or two notches are replaced by VO_2_). To present this description clearly, the corresponding structure sketch of inserting VO_2_ is shown in Fig. 6a. It should be noted that other structure parameters remain unchanged. In other words, the three-dimensional structure diagram in this case is very similar to that in Fig. 1a, which is not repeated here. It can be predicted that when VO_2_ has the property of insulator (or dielectric) phase (such as the conductivity of *σ* = 200 S/m), the surface structure of the metamaterial absorber can still be regarded as analogy I-typed resonator, thus it should have triple-band absorption characteristics. However, when VO_2_ exhibits the metal phase (for example, its conductivity *σ* is equal to 200,000 S/m), the surface structure can be considered as a complete rectangular patch, so it should have the absorption performance of rectangular patch, that is, dual-band absorption could be obtained. As revealed in Fig. 6b, it is observed that triple-band absorption can be realized when conductivity of VO_2_ *σ* is equal to 200,000 S/m, while dual-band absorption feature is achieved for the conductivity of *σ* = 200 S/m. These results are in line with the theoretical expectations. Additionally, Fig. 6c shows the absorption curves under different conductivity values, i.e. during the transition from dielectric phase to metal phase. With the increase (or decrease) of conductivity, the absorption performance of the designed metamaterial absorber changes gradually, which has the characteristics of adjustable.

(2) The second active modulation strategy is to introduce a continuous VO_2_ layer under the layer of the analogy I-typed resonator. Here the continuous VO_2_ layer has the thickness of 0.4 μm, which is larger than the skin depth of incident beam. In fact, larger thickness will get similar adjustment performance. In order to facilitate the research and avoid the repetition of similar simulation results, we only consider the case of 0.4 μm. Different from the active modulation obtained by the first strategy, the strategy designed here only introduces a continuous VO_2_ layer without changing the surface structure of the initial metamaterial absorber. To intuitively give the second active modulation strategy and avoid the repetition of front view of structure sketch, we only give its three-dimensional structure diagram, as illustrated in Fig. 7a. Because the thickness of VO_2_ is selected to be greater than the skin depth of incident electromagnetic wave, when VO_2_ has high conductivity of *σ* = 200,000 S/m and presents the metal phase, it can completely block the incident beam into the layers of dielectric sheet and bottom Au film, resulting in near-zero absorption. The red line in Fig. 7b clearly shows this resonance phenomenon. However, when VO_2_ possesses low conductivity of *σ* = 200 S/m and shows the insulator phase, it only acts as a dielectric sheet and does not affect the propagation of light, thus three resonance peaks with high absorption rates should be obtained. As seen in black line of Fig. 7b, three resonance peaks are indeed achieved. In addition, Fig. 7c gives the absorption curves of VO_2_ layer at different conductivity values during the insulator–metal phase transition. As revealed, the active modulation of absorption performance can be clearly found.

## Conclusion

In conclusion, we demonstrate a scheme to theoretically achieve triple-band near 100% absorption device using a simple metamaterial structure composed of an analogy I-typed Au resonator (i.e., a rectangular patch with two small notches) and a 14 μm thickness of dielectric sheet backed by a continuous Au board. Three discrete absorption frequency points operating at terahertz frequency with high absorption intensity are obtained. The three frequency points are all caused by localized resonances of the analogy I-typed resonator, while the near-field concentration positions of them on the analogy I-typed resonator are different. Influence of the structure parameters of the analogy I-typed resonator on the performance of the triple-band MBLA is analyzed. It is revealed that the number of absorption peaks can be strongly affected by the sizes of the small notch. We also present a kind of structure design based on a square Au patch with four same-sized notches to realize triple-band polarization-insensitive MBLA at terahertz frequency. Actively tunable absorption features could be further realized by employing two kinds of design strategies, and those two strategies are both based on the inserting or introducing vanadium dioxide with adjustable conductivity into the metamaterial structure. The transition from the insulator phase to metal phase of vanadium dioxide is the key factor to realize the modulation of absorption properties. These proposed triple-band MBLAs with simple structure designs, excellent resonant characteristics could have great application potentials in the field of terahertz technology.

## Data Availability

The data set used and/or analyzed in this study can be obtained from the corresponding author upon reasonable request.

## Abbreviations

MBLAs: Metamaterial based light absorbers
FWHM: Full width at half maximum
